# Draft genome sequence data of *Streptomyces* sp. SS1-1, an endophytic strain showing cytotoxicity against the human lung cancer A549 cell line

**DOI:** 10.1016/j.dib.2020.105497

**Published:** 2020-04-08

**Authors:** Quan Dang Nguyen, Phung Minh Truong, Thao Nguyen Thanh Vo, Truc Dao Xuan Chu, Chuong Hoang Nguyen

**Affiliations:** a Biotechnology Center of Ho Chi Minh City, Ho Chi Minh City, Vietnam; b Center for Research and Application in Bioscience, Ho Chi Minh City, Vietnam; c University of Science, Vietnam National University Ho Chi Minh City, Ho Chi Minh City, Vietnam

**Keywords:** Cytotoxicity, *Streptomyces*, NGS, A549 cell line, SS1-1

## Abstract

We report in this article the cytotoxicity of *Streptomyces* sp. SS1-1 against the human lung cancer A549 cell line, its draft genome sequence and a total of 20 predicted secondary metabolite biosynthetic gene clusters. *Streptomyces* sp. SS1-1 was an endophytic bacterial strain isolated from the plant *Catharanthus roseus* in Ho Chi Minh City, Vietnam. When cultured in the PY medium, this strain shows a cytotoxic effect on the A549 cell line. The draft genome of *Streptomyces* sp. SS1-1 has four contigs of total 7,815,656 bp and the GC content of this genome is 72.2%. AntiSMASH analysis reveals 20 putative biosynthetic gene clusters for the largest contig. The genome sequencing of *Streptomyces* sp. SS1-1 is essential for the molecular identification of gene cluster(s) responsible for secondary metabolite(s) with cytotoxic activity.

Specifications Table

**Table:** 

*Subject*	Biology
*Specific subject area*	Microbiology, Genomics, Biotechnology
*Type of data*	Figures, tables, draft genome sequence, raw sequencing data
*How data were acquired*	MTT (3-[4,5-methylthiazol-2-yl]−2,5-diphenyl-tetrazolium bromide) assay, flow cytometry analysis, genome sequencing by PacBio Sequel and Illumina HiSeq 4000
*Data format*	Raw and Analyzed
*Parameters for data collection*	Cytotoxic effect on the A549 cell line was evaluated by MTT assay and flow cytometry analysis. Genome sequencing of the strain was performed by PacBioSequel and Illumina HiSeq4000. Gene annotation and analysis were done by the PGAP, Busco, BBMap's MinHash Sketch programs. Putative biosynthetic gene clusters were predicted by the AntiSMASH program.
*Description of data collection*	*Streptomyces* sp. SS1-1 was isolated from *Catharanthus roseus* and cultured in the PY medium for expressing cytotoxic activity. MTT assay and flow cytometry analysis were performed on the A549 cell line to evaluate the cytotoxicity. Genomic DNA of *Streptomyces* sp. SS1-1 was extracted and sequenced by PacBio Sequel and HiSeq4000. The genome sequence was used for gene prediction and annotation as well as analysis to find putative biosynthetic gene clusters for secondary metabolites in *Streptomyces* sp. SS1-1.
*Data source location*	Center for Research and Application in Bioscience, Ho Chi Minh City, Vietnam
*Data accessibility*	Data are available within this article. The draft genome sequence of *Streptomyces* sp. SS1-1 is available in GenBank under the accession number NZ_WBXN00000000.1. The raw sequencing data are available in the Sequence Read Archive (SRA) database under the accession number SRX6989938 at https://www.ncbi.nlm.nih.gov/sra/SRX6989938[accn].

## Value of the data

• *Streptomyces* sp. SS1-1 isolated from *Catharanthus roseus* expresses the cytotoxic effect on the human lung cancer A549 cell line.
• The draft genome sequence data of *Streptomyces* sp. SS1-1 will be useful for finding biosynthetic gene cluster(s) responsible for secondary metabolite(s) with cytotoxic activity.
• In *Streptomyces* sp. SS1-1 genome, 20 putative biosynthetic gene clusters for secondary metabolites were identified.
• Data presented here could enrich the database of biosynthetic gene clusters responsible for cytotoxicity in *Streptomyces* bacteria.

## 1 Data

*Streptomyces* sp. SS1-1 was isolated from the stem of *Catharanthus roseus* using the protocol of Qin et al. with small modifications. No microbial growth was detected on the SFM agar and the nutrient agar when the surface-sterilized stem sample was imprinted on these culture media. Moreover, the spore of *Streptomyces* sp. SS1-1 could not grow on the SFM agar after sequential treatment with the solutions used for surface sterilization of the stem sample. These data supported the endophytic origin of *Streptomyces* sp. SS1-1. In literature, *Catharanthus roseus* was used to isolate the endophytic microorganisms including *Streptomyces* species which were associated with antibacterial, antifungal, anticancer activities [1], [2], [3], [4].

**Fig. 1:**
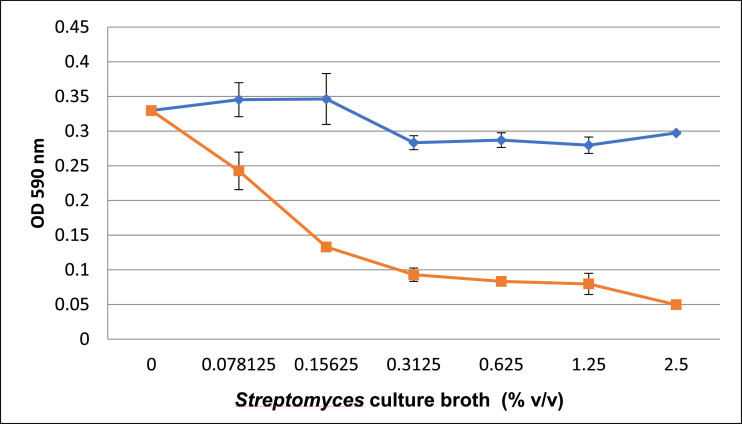
Cytotoxic activity of the *Streptomyces* sp. SS1-1 culture broth on the A549 cell line revealed by MTT assay. Curves show the 48 h of treatment of the A549 cells with the PY medium (the upper curve) and with the PY medium inoculated with *Streptomyces* sp. SS1-1 (the lower curve). Values were represented as mean  ±  SD (*n* = 3).

**Fig. 2:**
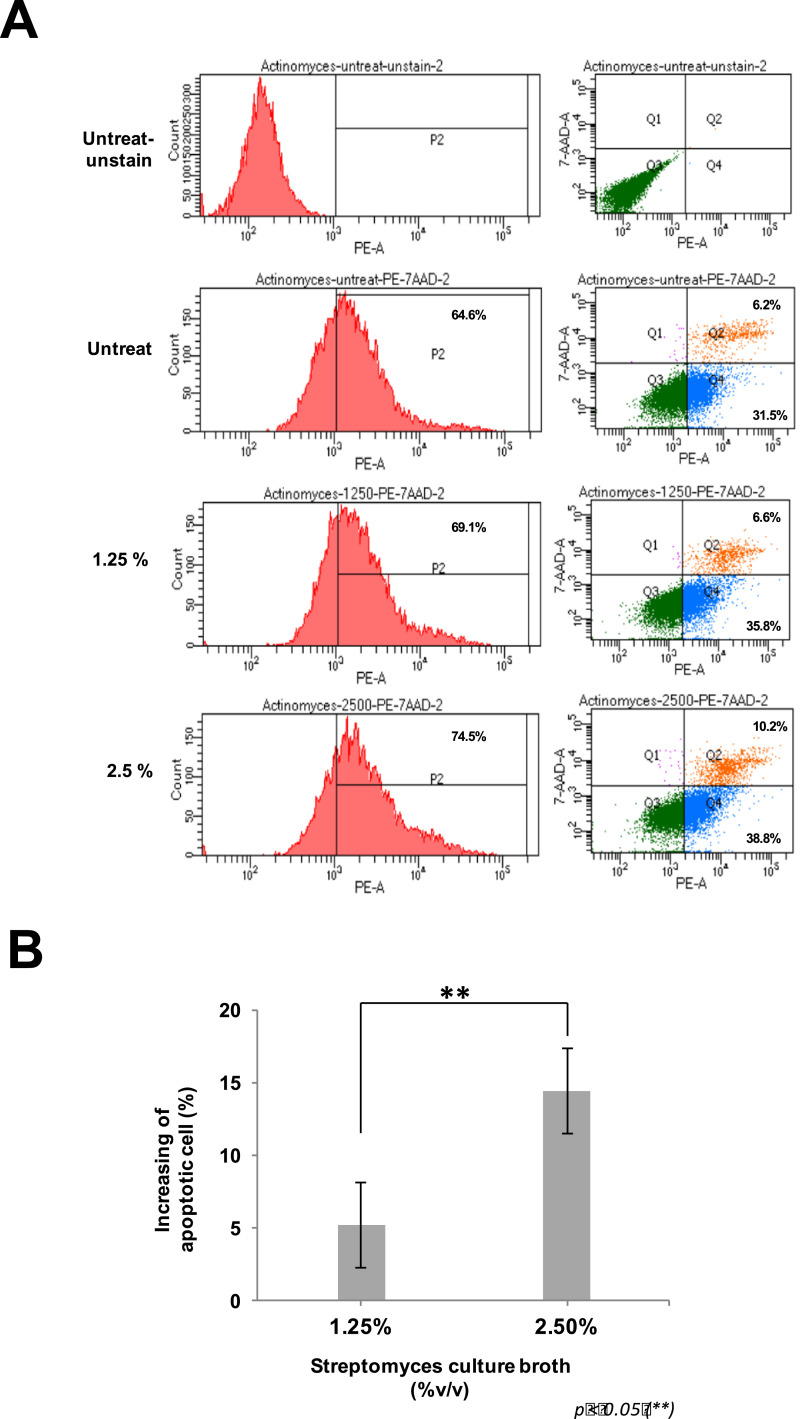
Flow cytometry analysis of the A549 cell apoptosis caused by the 24 h of treatment with the *Streptomyces* sp. SS1-1 culture broth. A: Representative flow cytometry result showing the apoptotic cells of the A549 cell line treated with 0%, 1.25% and 2.5% (v/v) of the *Streptomyces* sp. SS1-1 culture broth; B: Increasing of apoptotic cells treated with 1.25% and 2.5% (v/v) of the *Streptomyces* sp. SS1-1 culture broth compared with that of the untreated cells. Values were represented as mean  ±  SD (*n* = 3) with a *t*-test analysis using Microsoft Excel.

*Streptomyces* sp. SS1-1 was tested for cytotoxic activity on the human lung cancer A549 cell line. The PY culture broth inoculated with *Streptomyces* sp. SS1-1 showed a remarkable reduction in the A549 cell count in comparison with the unseeded PY culture broth revealed by the MTT (3-[4,5-methylthiazol-2-yl]−2,5-diphenyl-tetrazolium bromide) assay (Fig. 1). Moreover, cell apoptosis analyzed by flow cytometry showed a 5.23% increase of apoptotic cells when the A549 cell line was treated with 1.25% (v/v) of the *Streptomyces* sp. SS1-1 culture broth compared with that of the untreated cells. The apoptotic cell rate increased by up to 14.45% when the cancer cells were treated with 2.5% (v/v) of the *Streptomyces* sp. SS1-1 culture broth. The *Streptomyces* culture broth induced the increase of both early apoptotic cells (PE Annexin V positive, 7-AAD negative) and late apoptotic cells (PE Annexin V and 7-AAD positive) in the A549 cell population (Fig. 2). The preliminary data showed that the cytotoxic effect of *Streptomyces* sp. SS1-1 on the human lung cancer A549 cell line could be due to apoptosis but other cytotoxic mechanisms could not be excluded.

Genome sequencing was performed for *Streptomyces* sp. SS1-1 to identify putative gene clusters for secondary metabolites, one(s) of which could be responsible for the cytotoxic activity of the strain. The assembled genome of *Streptomyces* sp. SS1-1 produced four contigs of total length 7,815,656 bp and the coverage of 147-fold. The genome completeness was checked by Busco and was 95.3% complete, with 95.3% of the genome single copy and 0.0% duplicated. The GC content of the draft genome was 72.2%. An assessment of the assembly by BBMap's MinHash Sketch suggested it is most similar to *Streptomyces* sp. NRRL WC-3604. A total of 7205 genes was predicted in the genome of *Streptomyces* sp. SS1-1 by the NCBI Prokaryotic Genome Annotation Pipeline. Of 7205 genes, there are 6831 protein-coding genes, 68 tRNA genes, 3 ncRNA genes, 18 rRNA (5S (6), 16S (6), 23S (6)) genes and a total of 285 pseudogenes (Table 1).

**Table 1:** Features of the draft genome of *Streptomyces* sp. SS1-1.

Feature	*Streptomyces* sp. SS1-1
Source of isolation	*Catharanthus roseus*
Genome size (bp)	7,815,656
Contig	4 (14,134 bp; 49,440 bp; 199,786 bp; 7,552,296 bp)
GC content (%)	72.2
Gene total	7205
Protein coding sequences	6831
tRNA	68
rRNA	6 (5S), 6 (16S), 6 (23S)
ncRNA	3
Pseudogene	285

Prediction of putative gene clusters for secondary metabolites performed with the AntiSMASH program showed 20 putative gene clusters for secondary metabolites in the largest contig of 7,552,296 bp while the other three smaller contigs had no predicted gene cluster for secondary metabolites. Among 20 predicted gene clusters of *Streptomyces* sp. SS1-1, five gene clusters had 100% similarity with known gene clusters in *Streptomyces* species. They were the gene clusters for ectoine (with the ectoine gene cluster of *Streptomyces anulatus*), melanin (with the melanin gene cluster of *Streptomyces coelicolor* A3(2)), terpene (with the albaflavenone gene cluster of *Streptomyces coelicolor* A3(2)), terpene/butyrolactone (with the gamma-butyrolactone gene cluster of *Streptomyces acidiscabies*), terpene (with the isorenieratene gene cluster of *Streptomyces argillaceus*). Five gene clusters in *Streptomyces* sp. SS1-1 showing moderate to high similarities with known gene clusters in *Streptomyces* species were terpene (with hopene of *Streptomyces coelicolor* A3(2) with 92% similarity), siderophore (with desferrioxamine B of *Streptomyces coelicolor* A3(2) with 83% similarity), type 2 polyketide (with spore pigment of *Streptomyces avermitilis* with 75% similarity), melanin (with melanin of *Streptomyces avermitilis* with 57% similarity), and type 1 PKS (with hitachimycin of *Streptomyces scarisporus* with 54% similarity). These gene clusters in *Streptomyces* sp. SS1-1 shared most of the similar genes with known gene clusters in *Streptomyces* species (terpene, siderophore, type 2 polyketide gene clusters) or at least contained core biosynthetic genes and additional biosynthetic genes with known gene clusters in *Streptomyces* species (melanin, hitachimycin gene clusters). Six gene clusters in *Streptomyces* sp. SS1-1 for siderophores, NRPS/NRPS-like, type 3 PKS, type 1 PKS/NRPS, butyrolactone, bacteriocin (Table 2) had very low similarity with known gene clusters for secondary metabolites in *Streptomyces* species. In addition, they did not share core biosynthetic genes and additional biosynthetic genes with known gene clusters for secondary metabolites in *Streptomyces* species. There were three gene clusters for two terpenes and one bacteriocin in the genome of *Streptomyces* sp. SS1-1 which had no match with known gene clusters for secondary metabolites in bacteria and fungi in the database of the AntiSMASH program (Table 2). A search in literature for cytotoxic activity of ectoine, melanin, albaflavenone, gamma-butyrolactone, isorenieratene, hopene, desferrioxamine B, spore pigment, hitachimycin revealed that hitachimycin, ectoine, melanin possess cytotoxic effects on cancer cell lines and brine shrimp model [5], [6], [7]. Further experiments will be performed to identify the involvement of the potential biosynthetic gene clusters in the cytotoxic activity of *Streptomyces* sp. SS1-1.

**Table 2:** Putative gene clusters for secondary metabolites in the 7,552,296 bp contig of the *Streptomyces* sp. SS1-1 draft genome.

Number	Position	Type	Most similar known cluster	Type	Similarity (%)
1	1,36,466..246,539	Type 1 PKS	Hitachimycin (*Streptomyces scarisporus*)	Polyketide	54
2	686,910..706,180	Terpene	ND	ND	ND
3	1,301,113..1,319,856	Terpene	ND	ND	ND
4	1,605,024..1,615,422	Ectoine	Ectoine (*Streptomyces anulatus*)	Other	100
5	2,525,072..2,534,456	Melanin	Melanin (*Streptomyces coelicolor* A3(2))	Other	100
6	2,614,349..2,605,105	Siderophore	Desferrioxamine B (*Streptomyces coelicolor* A3(2))	Other	83
7	2,796,432..2,806,015	Butyrolactone	Fluostatin *(uncultured bacterium BAC AB649/1850)*	Type 2 PKS	12
8	4,772,131..4,792,445	Terpene	Albaflavenone (*Streptomyces coelicolor* A3(2))	Terpene	100
9	4,916,120..4,988,638	Type 2 PKS	Spore pigment (*Streptomyces avermitilis*)	Type 2 PKS	75
10	5,424,194..5,434,924	Siderophore	Ficellomycin (*Streptomyces ficellus)*	NRPS	3
11	5,501,459..5,585,717	NRPS, NRPS-like	Auroramycin (*Streptomyces filamentosus*)	Type 1 PKS	10
12	5,652,601..5,662,898	Bacteriocin	ND	ND	ND
13	5,695,869..5,716,543	Terpene, butyrolactone	Gamma-butyrolactone (*Streptomyces acidiscabies*)	Other	100
14	5,842,791..5,855,911	Siderophore	Grincamycin (*Streptomyces lusitanus*)	Type 2 PKS-saccharide	5
15	6,300,385..6,326,310	Terpene	Hopene (*Streptomyces coelicolor* A3(2))	Terpene	92
16	6,844,582..6,854,797	Bacteriocin	Informatipeptin (*Streptomyces viridochromogenes DSM 40,736)*	Lanthipeptide	42
17	6,886,817..6,897,191	Melanin	Melanin (*Streptomyces avermitilis*)	Other	57
18	6,940,607..7,019,736	Type 1 PKS, NRPS	Foxicins A-D (*Streptomyces diastatochromogenes)*	NRPS-Type 1 PKS	12
19	7,129,058..7,154,715	Terpene	Isorenieratene (*Streptomyces argillaceus)*	Terpene	100
20	7,278,601..7,319,785	Type 3 PKS	Lipopeptide 8D1-1/ Lipopeptide 8D1-1 (*Streptomyces rochei)*	NRPS	11

ND: not determined.

The draft genome sequence of *Streptomyces* sp. SS1-1 has been deposited in GenBank under the accession number NZ_WBXN00000000.1. The raw sequencing data were submitted to the SRA database under the accession number SRX6989938 at https://www.ncbi.nlm.nih.gov/sra/SRX6989938[accn].

## 2 Experimental design, materials and methods

For isolating *Streptomyces* sp. SS1-1 from *Catharanthus roseus*, the plant was collected, transported to the laboratory and processed immediately within 2–3 h. Stems were cut with a size of 3 cm and thoroughly washed in running tap water to remove epiphytes and other adhered materials on the surface of the stem. Then, surface sterilization of the stem sample was performed according to the protocol of Qin et al. [8] with modifications. Firstly, stems were rinsed with 0.1% Tween 20 for a few seconds and washed with sterile distilled water. Then, the stem sample was immersed in 5% NaOCl in 5 min and then transferred to 5% Na_2_S_2_O_3_ for 10 min to neutralize NaOCl. In the following steps, the stem sample was soaked in 70% ethanol for 5 min and then washed with sterile distilled water to remove residual ethanol. Finally, the stem sample was rinsed in 10% NaHCO_3_ for 10 min and allowed to air-dry in the cabinet. The stem sample was then ground in a mortar with sterile 0.9% NaCl and the plant extract was spread on the Soya Flour Mannitol (SFM) agar plate containing 30 μg/ml nalidixic acid, 50 μg/ml cycloheximide, and 50 μg/ml nystatin to suppress the growth of Gram-negative bacteria and fungi. The SFM plate was incubated at 28 °C for 7 days to promote the growth of *Streptomyces* species. For checking the effectiveness of the surface sterilization protocol, the surface-sterilized tissue was imprinted on the SFM agar and the nutrient agar to observe the microbial growth. Alternatively, the spore of *Streptomyces* sp. SS1-1 was sequentially treated with the disinfecting solutions as above and plated on SFM agar to check whether they can grow after the surface sterilization protocol.

For testing cytotoxicity activity, *Streptomyces* sp. SS1-1 was cultured in the PY broth [9] using baffled Erlenmeyer at 28 °C. The Erlenmeyer was shaken at 180 rpm for 5 days. Then, the cultured broth was filtered through a 0.45 μm filter unit and was used directly for the MTT assay and the flow cytometry analysis. For MTT assay, a quantity of 2 × 10^3^ A549 cells was distributed in each well of a 96-well plate containing 100 μl of DMEM (Dulbecco's modified Eagle's medium) supplemented with 10% FBS (Fetal bovine serum), 100 units/ml penicillin and 100 μg/ml streptomycin. The cells were cultured for 24 h at 37 °C in a humidified 5% CO_2_ incubator for attachment. After that, the cell culture medium was replaced with the fresh medium containing various concentrations of the PY broth and the PY broth inoculated with *Streptomyces* sp. SS1-1 and left for 48 h. Then, the medium was discarded and 100 μl of serum-free medium and 10 μl of 5 mg/ml MTT solution (Biobasic) were added into each well. After incubation at 37 °C for 3 h, the MTT medium was replaced by 100 µl of MTT solvent and the absorbance was read at 590 nm. For flow cytometry analysis, 1.5 × 10^5^ of A549 cell was seeded in each well of a 6-well plate containing 5 ml of DMEM supplemented with 10% FBS and the plate was incubated for 48 h. Then, the medium was replaced by 3 ml of fresh medium containing 0%, 1.25% and 2.5% (v/v) of the *Streptomyces* sp. SS1-1 culture broth and the plate was incubated for 24 h. Cells were detached from the plate and stained with PE Annexin V and 7-AAD (Apoptosis detection kit, BD) before cell apoptosis was detected by flow cytometry.

For genome sequencing using NGS technologies, the mycelium of *Streptomyces* sp. SS1-1 was harvested from 3 days old culture in Tryptone Soya Broth and subjected for genomic DNA extraction with the Qiagen MagAttract HMW kit (Qiagen). Genomic DNA was converted into sequencing libraries using the PacBio Multiplex kit (Pacific Biosciences, Menlo Park, CA) and subsequently, sequenced with a PacBio Sequel using Sequencing Reagent Kit v2.1 by the University of Oregon GC3F facility. In parallel, genomic DNA was converted to Illumina libraries using the Illumina Nextera DNA Flex kit (Illumina, San Diego, CA) and sequenced on a HiSeq4000 with paired-end 150 bp reads (University of Oregon GC3F facility). PacBio Sequel reads were assembled with Canu 1.7 [10] with a genome size of 8 Mbp and option corOutCoverage=60. The Canu assembly was polished with the PacBio raw reads using the arrow program from PacBio. This consensus was then polished using Pilon [11] and the Illumina reads.

Genome completeness of *Streptomyces* sp. SS1-1 was checked by BUSCO [12]. Genome sequence comparison was performed by BBMap's MinHash Sketch program. Gene prediction and annotation were carried out using the NCBI Prokaryotic Genome Annotation Pipeline [13]. AntiSMASH program [14] was used to search putative gene clusters for secondary metabolites in *Streptomyces* sp. SS1-1 genome.

## CRediT authorship contribution statement

**Quan Dang Nguyen:** Investigation, Writing - original draft, Writing - review & editing. **Phung Minh Truong:** Investigation. **Thao Nguyen Thanh Vo:** Investigation, Formal analysis. **Truc Dao Xuan Chu:** Investigation. **Chuong Hoang Nguyen:** Conceptualization, Supervision, Writing - original draft, Writing - review & editing.

## Declaration of Competing Interest

The authors declare that they have no known competing financial interests or personal relationships that could have appeared to influence the work reported in this paper.

## Appendix Supplementary materials

Image, application 1

Image, application 2
